# Shared genetic variance between obesity and white matter integrity in Mexican Americans

**DOI:** 10.3389/fgene.2015.00026

**Published:** 2015-02-13

**Authors:** Elena A. Spieker, Peter Kochunov, Laura M. Rowland, Emma Sprooten, Anderson M. Winkler, Rene L. Olvera, Laura Almasy, Ravi Duggirala, Peter T. Fox, John Blangero, David C. Glahn, Joanne E. Curran

**Affiliations:** ^1^Department of Family Medicine, Madigan Army Medical CenterTacoma, WA, USA; ^2^Department of Psychiatry, Maryland Psychiatric Research Center, University of Maryland School of MedicineBaltimore, MD, USA; ^3^Department of Physics, University of MarylandBaltimore, MD, USA; ^4^South Texas Diabetes and Obesity Institute, University of Texas Health Science Center at San AntonioSan Antonio, TX, USA; ^5^Department of Psychiatry, Yale UniversityNew Haven, CT, USA; ^6^Olin Neuropsychiatry Research Center, Institute of LivingHartford, CT, USA; ^7^Department of Clinical Neurosciences, Oxford Centre for Functional MRI of the Brain, University of OxfordOxford, UK; ^8^Department of Psychiatry, University of Texas Health Science Center at San AntonioSan Antonio, TX, USA; ^9^Research Imaging Institute, University of Texas Health Science Center at San AntonioSan Antonio, TX, USA

**Keywords:** diffusion tensor imaging, genotype, Mexican American, obesity, genetics, white matter, fractional anisotropy

## Abstract

Obesity is a chronic metabolic disorder that may also lead to reduced white matter integrity, potentially due to shared genetic risk factors. Genetic correlation analyses were conducted in a large cohort of Mexican American families in San Antonio (*N* = 761, 58% females, ages 18–81 years; 41.3 ± 14.5) from the Genetics of Brain Structure and Function Study. Shared genetic variance was calculated between measures of adiposity [(body mass index (BMI; kg/m^2^) and waist circumference (WC; in)] and whole-brain and regional measurements of cerebral white matter integrity (fractional anisotropy). Whole-brain average and regional fractional anisotropy values for 10 major white matter tracts were calculated from high angular resolution diffusion tensor imaging data (DTI; 1.7 × 1.7 × 3 mm; 55 directions). Additive genetic factors explained intersubject variance in BMI (heritability, *h*^2^ = 0.58), WC (*h*^2^ = 0.57), and FA (*h*^2^ = 0.49). FA shared significant portions of genetic variance with BMI in the genu (ρG = −0.25), body (ρG = −0.30), and splenium (ρG = −0.26) of the corpus callosum, internal capsule (ρG = −0.29), and thalamic radiation (ρG = −0.31) (all *p*'s = 0.043). The strongest evidence of shared variance was between BMI/WC and FA in the superior fronto-occipital fasciculus (ρG = −0.39, *p* = 0.020; ρG = −0.39, *p* = 0.030), which highlights region-specific variation in neural correlates of obesity. This may suggest that increase in obesity and reduced white matter integrity share common genetic risk factors.

## Introduction

Obesity is an immediate public health problem and the second leading cause of preventable death globally (Ogden et al., 2007). Prevalence of obesity is higher among Mexican Americans (29%) than non-Hispanic whites (21%) (Flegal et al., 2010) and among the Mexican American population, obesity is heritable (Comuzzie et al., 2000; Li et al., 2006). Not only does excess weight increase risk for a cascade of physical (Kawachi, 1999) and psychological morbidities (Kawachi, 1999), obesity is associated with cognitive impairments and reduced integrity of cerebral tissue, especially in cerebral white matter (Ward et al., 2005; Taki et al., 2008; Marks et al., 2010; Kazlouski et al., 2011; Stanek et al., 2011). Adequate brain function relies on the structural integrity of cerebral white matter, which is responsible for maintenance of normal connectivity among regions throughout the brain. Obesity is associated with white matter hyperintensities (Jagust et al., 2005) and neurochemical alterations (Gazdzinski et al., 2008), in addition to differences in white matter volumes (Jagust et al., 2005; Raji et al., 2010; Walther et al., 2010). Some studies observed gray and white matter volume abnormalities of the frontal (Walther et al., 2010), temporal (Haltia et al., 2007; Walther et al., 2010), and parietal (Walther et al., 2010) lobes that show partial improvement following diet-induced weight reduction (Haltia et al., 2007). Reduced white matter volumes have also been reported in the frontal lobes, anterior cingulum, and corona radiata among obese older adults compared to lean participants (Raji et al., 2010; Walther et al., 2010). Findings of macrostructural white matter abnormalities in obesity are inconsistent, leading to investigations of microstructural architecture of white matter tracts using more sophisticated imaging modalities.

Diffusion tensor imaging (DTI) is a fully quantitative technique capable of ascertaining subtle decline in white matter integrity (Maclullich et al., 2009). DTI is most frequently used to assess microstructural integrity of white matter, as it has an advantage over standard anatomical imaging techniques since it is sensitive to subtle white matter damage that precedes atrophic changes (e.g., decline in regional white matter volumes) (Maclullich et al., 2009). The most frequently used index of tract architecture is fractional anisotropy, an index of the preferential restriction of water diffusion in directions perpendicular to the main fibers as opposed to parallel to them. Inverse associations of BMI and fractional anisotropy have been reported in the right posterior cingulum among healthy older adults (Marks et al., 2010), and in the corpus callosum among obese healthy adults (Stanek et al., 2011; Xu et al., 2013). Significant contributions of genetic factors on fractional anisotropy have been found for most major white matter tracts (Kochunov et al., 2010, 2014; Jahanshad et al., 2013) including in the splenium and genu of the corpus callosum and the superior longitudinal fasciculus bilaterally in twins (Pfefferbaum et al., 2001; Chiang et al., 2009), but the relationship between BMI and diffusivity was not examined in these studies. Taken together, DTI studies examining neural correlates of obesity and twin studies reporting genetic contributions to fractional anisotropy provide initial evidence of obesity-associated deficits in white matter microstructure. Studies have separately identified factors that contribute to phenotypic variation in white matter integrity (Chiang et al., 2011) and obesity (Hasselbalch, 2010). Without studies examining genetic contributions on fractional anisotropy in family cohorts of varied BMI, this preliminary evidence does not allow for interpretation of shared genetic variance between white matter and adiposity.

One way to uncover additional information about the relationship between white matter and BMI is by examining shared genetic factors that influence these traits. An estimated 65% of variation in obesity is familial or genetic (Nan et al., 2012) and large family cohorts are ideal to study shared genetic risk factors. Previous studies have identified genes that are significantly associated with an increased risk of obesity (Greenfield et al., 2003; Dina et al., 2007; Cauchi et al., 2009; McCaffery et al., 2009; Li et al., 2010; Qi et al., 2014) and factors that contribute to phenotypic variation in white matter integrity (Chiang et al., 2011) and adiposity (Hasselbalch, 2010).

Some diseases may present differently in different tissues, termed as pleiotropic effects of the same gene. There is evidence of pleiotropic effects of genetic or environmental factors contributing to obesity in Mexican Americans (Comuzzie et al., 2000; Arya et al., 2004) and obesity-associated white matter deficits have been reported using DTI in other populations (Marks et al., 2010; Kazlouski et al., 2011; Stanek et al., 2011). Detailed examinations of individuals with specific genes may reveal different presentations of BMI and/or weight-related disease, some that may be genetic and others that may be environmentally induced. However, the majority of information on obesity-associated white matter deficits has been acquired without the use of genetic approaches (Marks et al., 2010; Kazlouski et al., 2011; Stanek et al., 2011). Examination of the genetic correlations between obesity measures and white matter integrity has not yet been reported. Understanding the influence of shared genetic factors to phenotypic variation in white matter deficits and obesity can potentially aid in the development of more focused preventive and therapeutic strategies. Ultimately, knowing genetic and environmental factors that are involved in traits, such as BMI, can assist clinicians in ruling out common and environmental causes of conditions that may be caused by a heritable defect.

Since little is known about the shared genetic and environmental influences on obesity and white matter integrity, we assessed the shared genetic variance between adiposity and fractional anisotropy in a cohort of Mexican American individuals from large extended families (Mitchell et al., 1996). We used bivariate genetic correlation analyses to calculate the proportion of shared genetic variance between BMI and waist circumference with fractional anisotropy (Almasy and Blangero, 1998). Body mass index (BMI; kg/m^2^) and waist circumference (WC; in) are frequently used as measures of obesity risk (Frayling et al., 2007; Rask-Andersen et al., 2011). In most people BMI correlates with the amount of fat present (World Health Organization, 2000) and inverse associations of BMI and fractional anisotropy have been reported (Marks et al., 2010). WC is a better indicator of central adiposity as it is associated with an increased disease risk and may suggest more severe physical and neural deficits. It is recommended that BMI and WC be used in tandem for clinical examination (Ferrannini et al., 2008). Average fractional anisotropy across the white matter skeleton has been shown to be heritable in multiple cohorts (Jahanshad et al., 2013) and was obtained using tract-based spatial statistics (TBSS). This was selected as the primary phenotype to assess the influence of genetic and environmental factors on weight-related fractional anisotropy impairments. We hypothesized that genetic factors associated with obesity were also associated with reduced white matter fractional anisotropy.

## Materials and methods

Analyses were performed using participants from the Genetics of Brain Structure and Function Study (Olvera et al., 2011), for whom the DTI, BMI, and WC measurements on the day of the imaging were available. Subjects were excluded for MRI contraindications, history of neurological illnesses (*n* = 5), or stroke (*n* = 14), transient ischemic attack or other major neurological event (*n* = 3). The participants in the study were urban-dwelling Mexican Americans from large extended pedigrees selected randomly from the San Antonio community. These subjects are characterized by a relatively adverse body weight profile, including increased rates of obesity, dyslipidemia, glucose intolerance, and hyperinsulinemia, when compared with non-Hispanic whites in San Antonio (Olvera et al., 2011). Additional recruitment details of the sample are available elsewhere (Olvera et al., 2011; Glahn et al., 2012). All subjects provided written informed consent on forms approved by the Institutional Review Board of the University of Texas Health Science Center at San Antonio (UTHSCSA).

### BMI and WC measurement

BMI is a clinical measure of obesity based on body weight scaled to height. In most people BMI correlates with the amount of fat present (World Health Organization, 2000). WC is a measure of central obesity that indexes relative risk for metabolic diseases without requiring height. WC is a better indicator of central adiposity as it is associated with an increased disease risk and may suggest more severe physical and neural deficits. WC is typically measured while standing with arms hanging down loosely, at the level of the abdomen. The National Heart, Lung, and Blood Institute (NHLBI) has adopted values of WC >102 cm (40 inches) in men and >88 cm (35 inches) in women as meeting criteria for increased metabolic syndrome risk. WC measurements are highly reproducible for both men and women (e.g., *r* = 0.998 at the iliac crest site). Still, the measurement site that best correlates to disease risk has yet to be established.

### Diffusion tensor imaging and processing

Diffusion tensor imaging was performed at the Research Imaging Institute, UTHSCSA, on a Siemens 3T Trio scanner equipped with a phase-array head coil. A single-shot, single refocusing spin-echo, echo-planar imaging sequence was used to acquire diffusion-weighted data with a spatial resolution of 1.7 × 1.7 × 3.0 mm. The sequence parameters were: TE/TR = 87/8000 ms, FOV = 200 mm, 55 isotropically distributed diffusion weighted directions, two diffusion weighting values, *b* = 0 and 700 s/mm^2^ and three *b* = 0 (non-diffusion-weighted) images. These parameters were calculated using an optimization technique that maximizes the contrast to noise ratio for fractional anisotropy measurements (Jones et al., 1999).

Details for the processing of DTI scans are described elsewhere (Kochunov et al., 2010, 2012). In short, the tract-based spatial statistics (TBSS) software (Smith et al., 2006) as part of FSL (http://fsl.fmrib.ox.ac.uk/fsl/fsl4.0/tbss/index) was used for multi-subject analysis of fractional anisotropy images. Fractional anisotropy images were created by fitting the diffusion tensor to the raw diffusion data. All fractional anisotropy images were non-linearly aligned to a group-wise, minimal-deformation target (MDT) brain. Next, individual fractional anisotropy images were averaged to produce a group-average anisotropy image. This image is used to create a group-wise skeleton of white matter tracts that encodes the medial trajectory of the white matter fiber-tracts. Finally, fractional anisotropy values from each image were projected onto the group-wise skeleton of white matter structures. This step accounts for residual misalignment among individual white matter tracts. Fractional anisotropy values are assigned to each point along a skeleton using the peak value found within a 20 mm distance perpendicular to the skeleton inversely weighted by their distance from the template skeleton. By assigning the peak value to the skeleton, this procedure effectively maps the center of individual white matter tracts on the skeleton.

The whole-brain average fractional anisotropy value for each subject was calculated as the average fractional anisotropy value for the entire white matter skeleton of about 300 × 10^3^ voxels. Next, the tract-wise average fractional anisotropy measurements were calculated for 10 major white matter tracts as described in our previous publications (Kochunov et al., 2010, 2012). The population-based, 3D, DTI cerebral white matter tract atlas developed in John Hopkins University (JHU) and distributed with the FSL package (Smith et al., 2006) was used to calculate population average diffusion parameter values along the spatial course of 10 white matter tracts: corpus callosum sub-regions (genu, body, splenium), the corona radiata, cingulum, external and internal capsule, posterior thalamic radiation, superior longitudinal fasciculus, and fronto-occipital fasciculus. The JHU atlas was non-linearly aligned to the MDT brain and image containing labels for individual tracts was transferred to MDT space using nearest-neighbor interpolation. Per-tract average values were calculated by averaging the values along the tracts in both hemispheres.

### Demographic analyses

Demographics and analyses of fractional anisotropy were conducted using the Statistical Package for the Social Sciences (SPSS) version 22.0 software package. All statistical tests were two-tailed with α < 0.05 unless otherwise stated.

Analyses were conducted on 761 participants (58% female) between the ages of 18 and 81 years (*M* = 41.3, *SD* = 14.5), all with BMI and WC data. Participants were categorized according to BMI into underweight (BMI <18.5 kg/m^2^; *n* = 10), healthy-weight (BMI 18.5–24.9 kg/m^2^; *n* = 128), overweight (BMI 25–29.9 kg/m^2^; *n* = 237) and obese (BMI = 30 kg/m^2^; *n* = 386) to assess fractional anisotropy differences between current clinical classifications for adiposity.

### Quantitative genetic analyses

Heritability of each imaging and obesity-related trait was estimated using SOLAR (Sequential Oligogenic Linkage Analysis Routines: http://solar.txbiomedgenetics.org (Almasy and Blangero, 1998). SOLAR uses variance component methods to analyze family-based quantitative data by partitioning the observed covariance into genetic and environmental components. Heritability (*h*^2^) is defined as the proportion of total phenotypic variance that is explained by additive genetic factors. An inverse-normal transformation was applied prior to variance decomposition analysis.

The difference in magnitude of shared genetic variance between BMI and WC with fractional anisotropy was calculated using bivariate genetic correlation analysis methods, also implemented in SOLAR. Bivariate genetic correlation analysis is performed to calculate the proportion of common genetic variance that influences both adiposity and white matter integrity. If the genetic correlation coefficient (ρG) is significantly different from zero, then a significant portion of the variability in the two traits is considered to be influenced by shared genetic factors (Almasy et al., 1997). Analyses were performed to study the genetic overlap between whole-brain and regional tract-wise fractional anisotropy values and adiposity measurements (BMI, WC). First, the magnitude of shared genetic effect between BMI and fractional anisotropy values was analyzed. This was repeated with the substitution of WC for BMI. Regional calculations were subsequently conducted for each tract and for corpus callosum components (genu, body, splenium). Bivarate genetic correlations between tract-wise regional fractional anisotropy values and adiposity measures (BMI, WC) were calculated separately. *P*-values were considered significant under a false-discovery rate (FDR) <5% across the 10 tracts of interest, and are reported under the lowest FDR at which they remain significant. The standard Benjamini–Hochberg method was used to control the FDR and increase the chance of identifying all the differentially expressed genes/traits (Benjamini and Hochberg, 1995).

All genetic analyses were conducted with age, sex, age × sex, age^2^, age^2^ × sex included as covariates. The covariates were chosen based on our prior findings of a quadratic (inverse-U) trajectory for FA and other neuroimaging measurements (Kochunov et al., 2011a, 2012; McKay et al., 2014). Health status was accounted for but was removed from the final models as it did not significantly impact results. Additional details of the bivariate correlation analysis are described elsewhere (Kochunov et al., 2011b).

## Results

### Demographics

Table 1 presents the sample characteristics. Overall, more than half (51%) of the sample was obese (BMI ≥ 30 kg/m^2^), one-third (31%) of the sample was overweight, 17% of the sample were healthy-weight and 1% of the sample was underweight. Underweight participants (*n* = 10) were 28.3 years old on average (*SD* = 10.9) with mean BMI of 17.4 kg/m^2^ (*SD* = 0.84) and WC of 29.4 in (*SD* = 3.5). No demographic variables significantly differed between the underweight and healthy-weight groups (*p*'s > 0.05) and underweight and healthy-weight categories were combined for all subsequent analyses. Mean BMI of the sample was 30.6 (*SD* = 6.4) kg/m^2^, average WC was 39.8 (*SD* = 6.0) in, and 42% (*n* = 320) of the sample was male. Overall, males had a significantly lower mean BMI (*M* = 29.9, *SD* = 6.1) than females (*M* = 31.1, *SD* = 6.6) [*t*_(759)_ = 2.602, *p* = 0.009] and WC was significantly lower among females (*M* = 39.3, *SD* = 5.9) than males (*M* = 40.5, *SD* = 6.1). There were significantly more obese females than males [χ^2^_(2)_ = 14.43, *p* = 0.001]. Between healthy-weight, overweight, and obese groups there were no significant differences in employment or education (all *p*'s > 0.05). Healthy-weight participants were significantly younger (*M* = 35.4, *SD* = 15.3) than participants in the overweight (*M* = 43.3, *SD* = 14.8) and obese (*M* = 42.2, *SD* = 13.5) groups, [*F*_(2, 758)_ = 14.88, *p* < 0.001].

**Table 1:** **Sample demographics (*N* = 761)**.

	**Total *n* = 761**	**Normal weight *n* = 138**	**Overweight *n* = 237**	**Obese *n* = 386**
**AGE (YEARS)**	41.3 ± 14.5	35.4 ± 15.3	43.3 ± 14.8	42.2 ± 13.5
**MEASURES OF OBESITY**				
BMI	30.6 ± 6.4	22.1 ± 2.1	27.4 ± 1.4	35.7 ± 4.5
WC	39.8 ± 6.0	32.6 ± 3.3	37.3 ± 3.8	43.8 ± 4.5
**SEX**				
Male	*n* = 320; 42.0%	*n* = 61; 8.0%	*n* = 121; 15.9%	*n* = 138; 18.1%
Female	*n* = 441; 58.0%	*n* = 77; 10.1%	*n* = 116; 15.2%	*n* = 248; 32.6%
**STATUS**				
Employed	*n* = 491; 64.5%	*n* = 82; 10.8%	*n* = 160; 21.0%	*n* = 249; 32.7%
Education (years)	12.0 ± 3.0	11.9 ± 2.7	12.0 ± 3.3	12.1± 2.9
**HEALTH STATUS**				
Heart disease	*n* = 17; 2.2%	*n* = 3;0.04%	*n* = 5;0.07%	*n* = 9; 1.2%
Diabetes	*n* = 104; 13.7%	*n* = 9; 1.2%	*n* = 29; 3.8%	*n* = 66; 8.7%
Hypertension	*n* = 190; 25.0%	*n* = 17; 2.2%	*n* = 47; 6.2%	*n* = 126; 16.6%

*BMI, body mass index (kg/m^2^); WC, waist circumference (in). Mean ± SD; n (%)*.

Whole-brain fractional anisotropy values did not differ between healthy-weight (*M* = 0.535, *SD* = 0.03), overweight (*M* = 0.532, *SD* = 0.03) or obese (*M* = 0.529, *SD* = 0.03) groups [*F*_(2, 758)_ = 14.88, *p* < 0.001].

#### Fractional anisotropy across BMI groups

Controlling for age and sex, analysis of covariance (ANCOVA) showed a significant difference in fractional anisotropy values in the SFO [*F*_(2, 754)_ = 4.29, *p* = 0.014]. Bonferroni-corrected *post-hoc* tests revealed significantly higher values in the SFO among healthy-weight than obese individuals, *p* = 0.014, *d* = 0.46. There were no other significant differences in fractional anisotropy values between weight groups.

#### White matter integrity by BMI, age, and gender

Partial correlations suggest that higher BMI was significantly associated with lower fractional anisotropy values globally and in all regions studied, though coefficients were small (*r* = −0.047 to −0.145, *p* = 0.05 to <0.001). WC was negatively correlated with all regions (*r* = −0.077 to −0.142, *p* = 0.04 to < 0.001) except the splenium and cingulum nor with global anisotropy values.

### Heritability analyses

Additive genetic factors explained a significant proportion of the intersubject variance in BMI (heritability, *h*^2^ = 0.58; *p* = 1 × 10^−25^) and WC (*h*^2^ = 0.57; *p* = 1 × 10^−27^) (Table 2). Age and age^2^ were significant covariates for BMI and WC. Sex also significantly covaried with WC, but not BMI. Together, covariates explained 4.9 and 5.4% of variance in BMI and WC, respectively.

**Table 2:** **Heritability estimates for BMI, WC and fractional anisotropy values, all values significant at *p* ≤ 0.001**.

**Trait**	***h*^2^ (*p*)**
BMI	0.58 (1E-25)^a,c^
Waist circumference	0.57 (1E-27)^a,b,c^
Average FA	0.49 (1E-12)^a,c^
Genu of corpus callosum FA	0.43 (1E-11)^a^
Body of corpus callosum FA	0.54 (1E-7)^a^
Splenium of corpus callosum FA	0.52 (1E-14)^a^
Corona radiata (CR)	0.49 (1E-10)^a^
Cingulum	0.47 (1E-11)^a^
External capsule (EC)	0.49 (1E-11)^a^
Internal capsule (IC)	0.45 (1E-9)^a^
Thalamic radiation (TR)	0.42 (1E-10)^a^
Superior fronto-occipital fasciculus (SFO)	0.41 (1E-9)^a^
Superior longitudinal fasciculus (SLF)	0.60 (1E-16)^a^

*FA, fractional anisotropy*.

*The pattern of significant (p ≤ 0.001) covariates is coded as age (^a^), sex (^b^), age^2^ (^c^)*.

Whole-brain (*h*^2^ = 0.49; *p* = 1 × 10^−12^) and regional (*h*^2^ = 0.41–0.60; *p*'s 1 × 10^−7^ to 1 × 10^−16^) fractional anisotropy values were similarly significantly heritable (Table 2).

### Bivariate genetic correlation analyses

**Figure 1:**
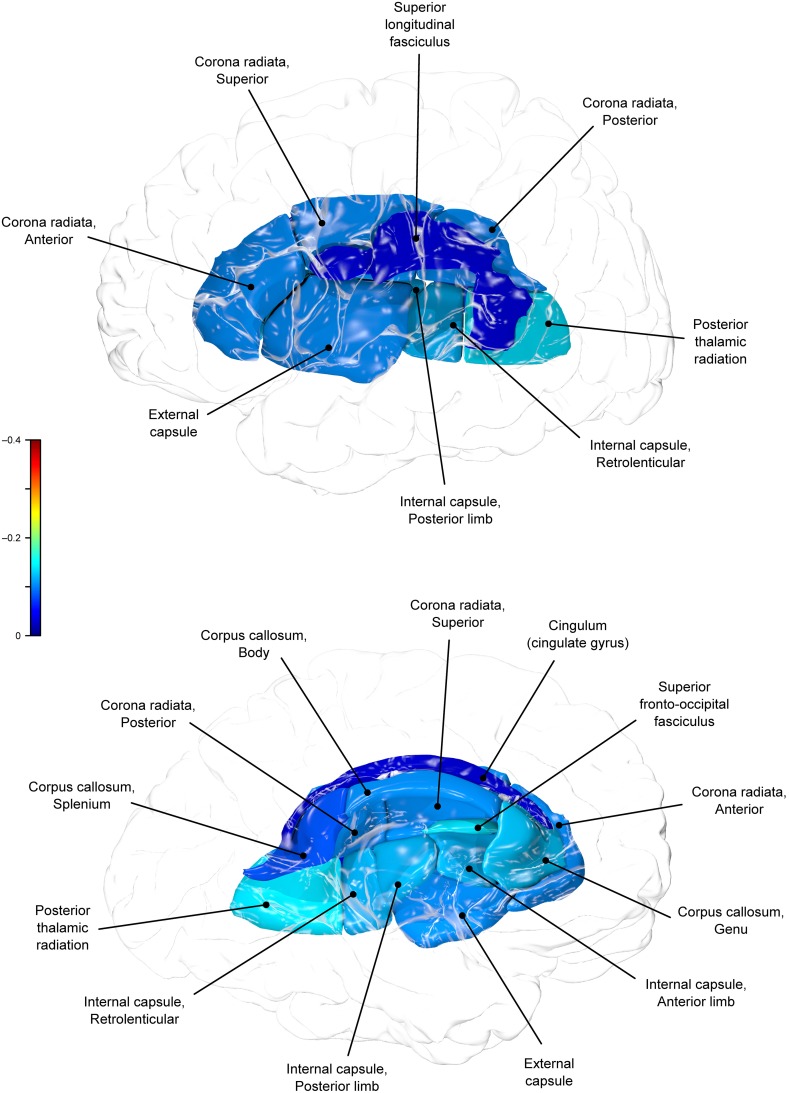
**Phenotypic (ρP) correlations between BMI and fractional anisotropy values**.

**Figure 2:**
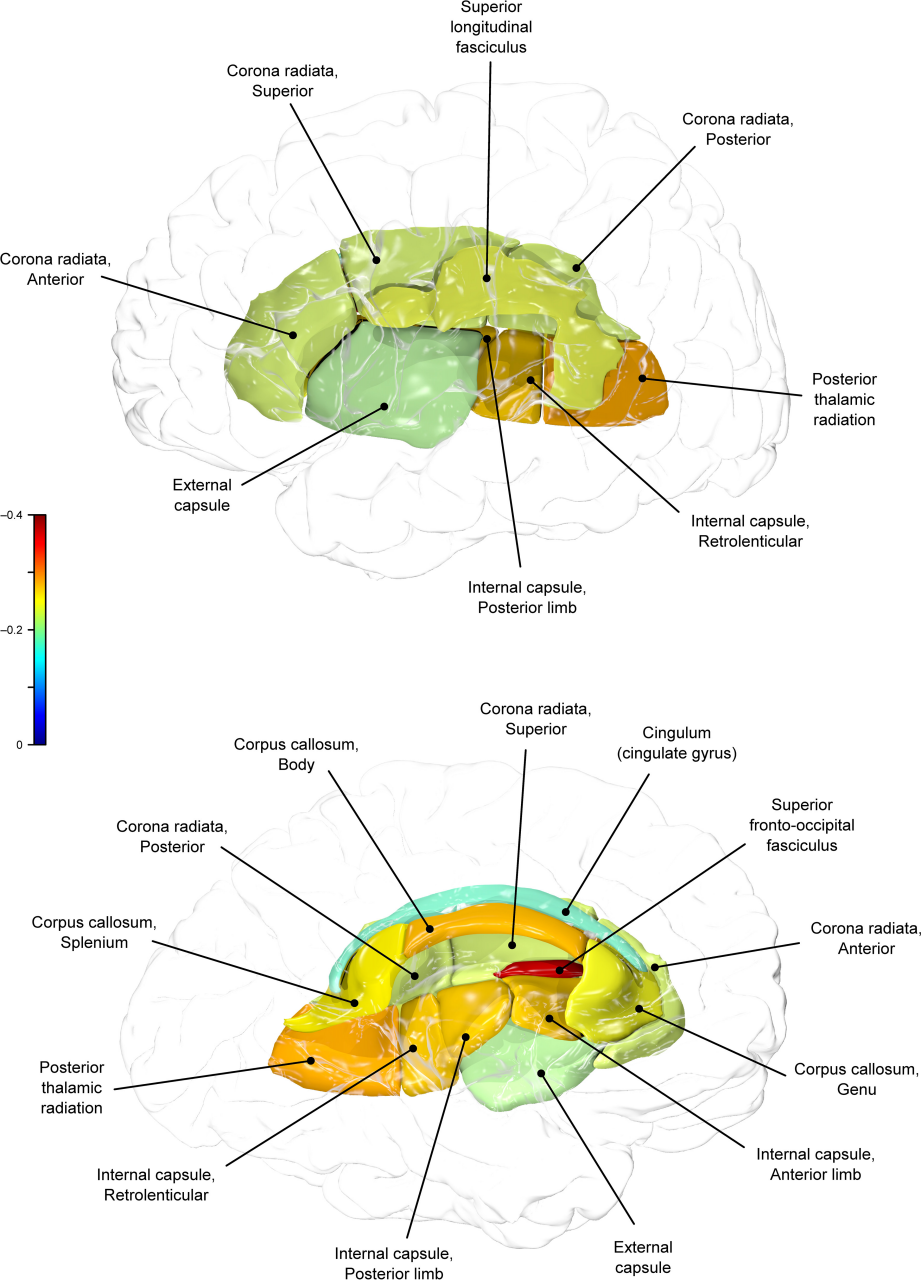
**Genetic (ρG) correlations between BMI and fractional anisotropy values**.

**Figure 3:**
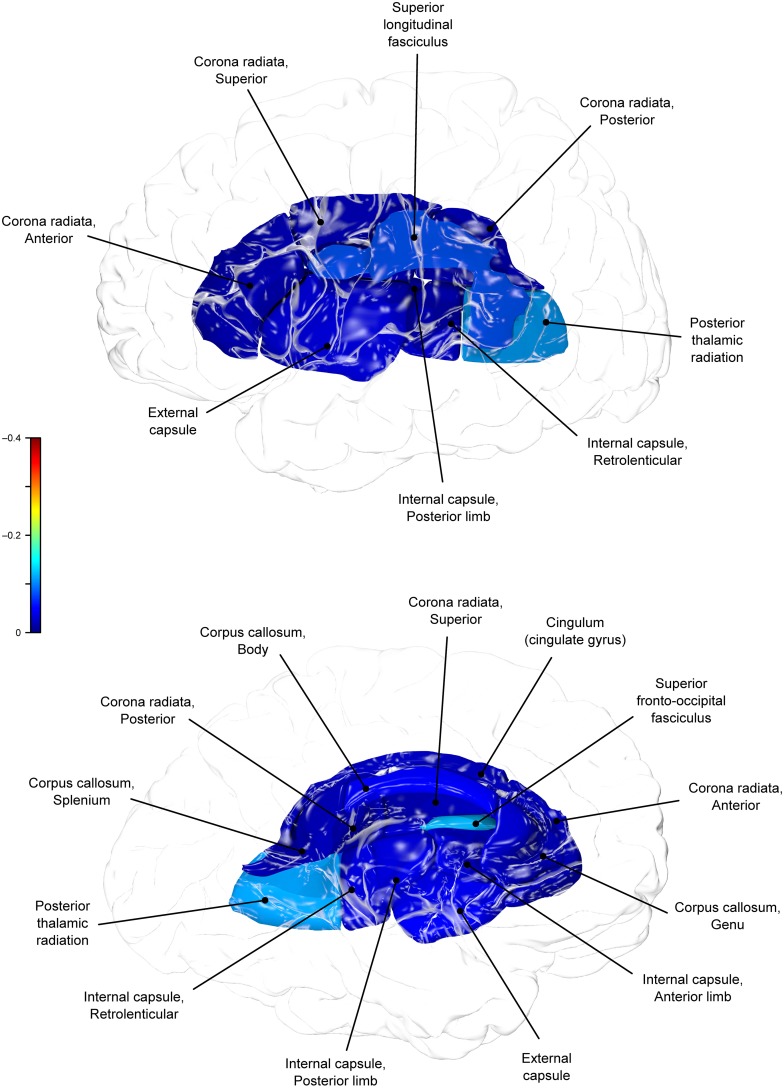
**Phenotypic (ρP) correlations between WC and fractional anisotropy values**.

**Figure 4:**
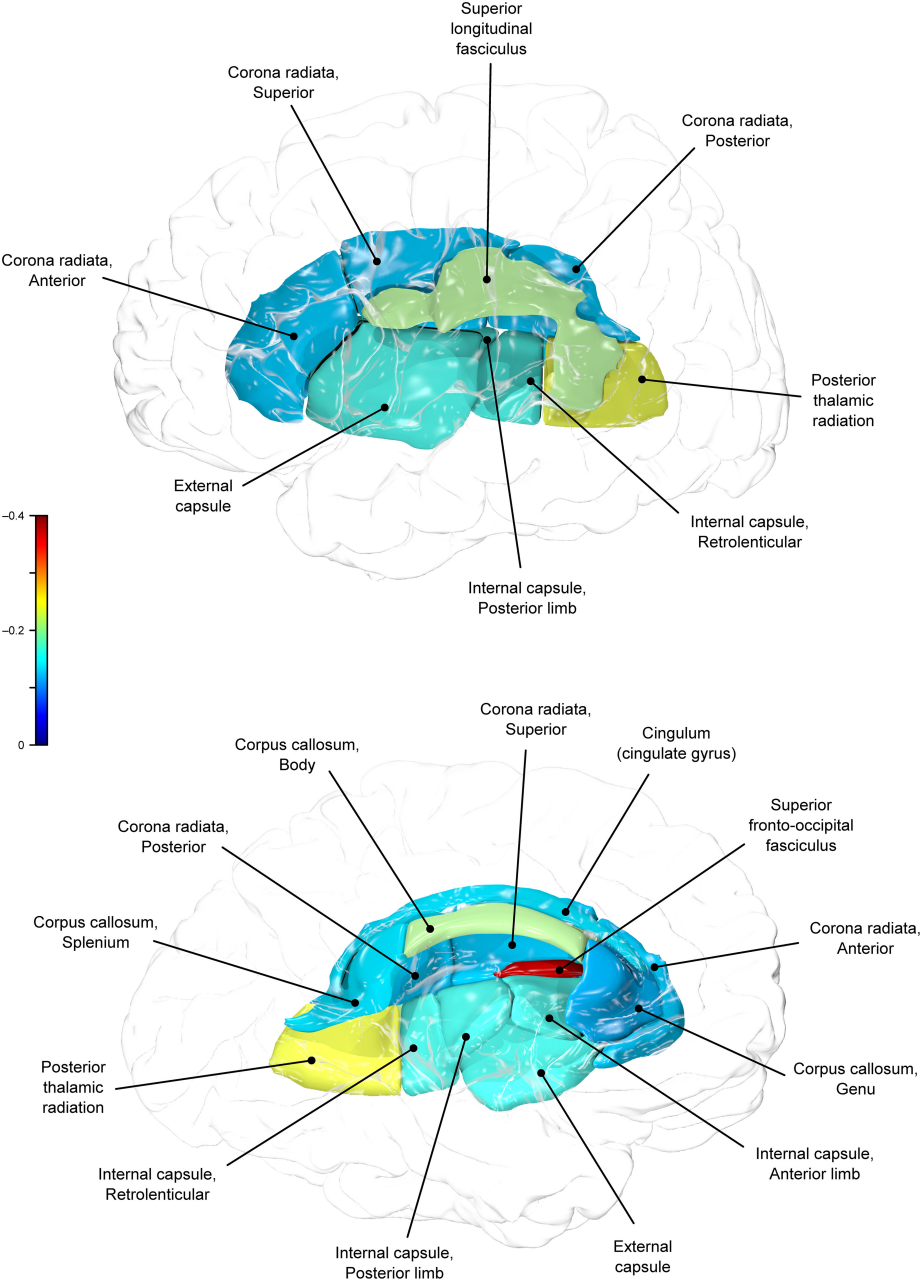
**Genetic (ρG) correlations between WC and fractional anisotropy values**.

Genetic overlap between BMI/WC and whole-brain/regional fractional anisotropy values was calculated using bivariate genetic correlation analysis. Whole brain fractional anisotropy values shared significant genetic variance with BMI (ρG = −0.25, *p* = 0.032) but not WC (ρG = −0.15, *p* = 0.194). By-tract genetic correlation analyses between BMI/WC and fractional anisotropy values indicated statistically significant (*p* = 0.05) genetic correlations with BMI in six of the 10 tracts studied and with WC in one tract but no environmental correlations between fractional anisotropy and either index of adiposity. Significant region-specific correlations between BMI/WC and fractional anisotropy are reported below. Phenotypic, genetic, and environmental correlations are provided in Table 3. The strength of phenotypic and genetic correlations for BMI and WC appear in Figures 1–4.

**Table 3:** **Phenotypic (ρP), genetic (ρG), and environmental (ρE) correlations between BMI, WC, and fractional anisotropy values**.

**Traits**	**ρP**	***p* (ρP)**	**ρG**	***p* (ρG)**	**ρE**	***p* (ρE)**	***p* (ρP) FDR**	***p* (ρG) FDR**	***p* (ρE) FDR**
**BODY MASS INDEX**									
Average FA	−0.06	0.108	−0.25	0.032^*^	0.16	0.145			
Genu of CC	−0.11	0.005^*^	−0.25	0.026^*^	0.08	0.488	0.017^*^	0.043^*^	0.542
Body of CC	−0.08	0.007^*^	−0.30	0.017^*^	0.09	0.351	0.018^*^	0.043^*^	0.542
Splenium of CC	−0.06	0.104	−0.26	0.026^*^	0.17	0.112	0.13	0.043^*^	0.542
Corona radiata	−0.08	0.050^*^	−0.23	0.066	0.11	0.397	0.071	0.079	0.542
Cingulum	−0.02	0.529	−0.18	0.119	0.14	0.175	0.529	0.119	0.542
External capsule	−0.08	0.043^*^	−0.21	0.067	0.08	0.456	0.071	0.079	0.542
Internal capsule	−0.10	0.018^*^	−0.29	0.024^*^	0.11	0.299	0.036^*^	0.043^*^	0.542
Thalamic radiation	−0.13	0.001^*^	−0.31	0.014^*^	0.06	0.562	0.005^*^	0.043^*^	0.562
SFO	−0.13	6.0 × 10^−4^^*^	−0.39	0.002^*^	0.12	0.259	0.005^*^	0.020^*^	0.542
SLF	−0.03	0.379	−0.24	0.071	0.16	0.115	0.421	0.079	0.542
**WAIST CIRCUMFERENCE**									
Average	−0.00	0.901	−0.15	0.194	0.19	0.101			
Genu	−0.02	0.534	−0.10	0.408	0.07	0.565	0.763	0.408	0.628
Body	−0.05	0.13	−0.21	0.112	0.09	0.411	0340	0.262	0.514
Splenium	−0.00	0.999	−0.13	0.276	0.16	0.167	0.999	0.333	0.514
Corona radiata	−0.01	0.853	−0.112	0.131	0.11	0.369	0.948	0.262	0.514
Cingulum	−0.01	0.821	−0.13	0.30	0.16	0.146	0.948	0.333	0.514
External capsule	−0.04	0.323	−0.16	0.179	0.10	0.357	0.646	0.298	0.514
Internal capsule	−0.03	0.513	−0.16	0.234	0.11	0.31	0.763	0.333	0.514
Thalamic radiation	−0.09	0.017^*^	−0.24	0.071	0.05	0.652	0.085	0.237	0.652
SFO	−0.10	0.010^*^	−0.39	0.003^*^	0.18	0.11	0.085	0.030^*^	0.514
SLF	−0.06	0.136	−0.21	0.064	0.15	0.222	0.340	0.237	0.514

*FA, fractional anisotropy; CC, Corpus callosum; SFO, superior fronto-occipital fasciculus; SLF, superior longitudinal fasciculus*.

* p < 0.05

BMI shared a significant proportion of genetic variance with fractional anisotropy in the genu (*r* = −0.11, *p* = 0.017; ρG = −0.25, *p* = 0.043; ρE = 0.08, *p* > 0.05), the body (*r* = −0.08, *p* = 0.018; ρG = −0.30; *p* = 0.043; ρE = 0.09, *p* > 0.05), and the splenium (*r* = −0.06, *p* = 0.13; ρG = −0.26, *p* = 0.043; ρE = 0.17, *p* > 0.05) of the corpus callosum. Genetic relationships between BMI and fractional anisotropy were also observed in the internal capsule (*r* = −0.10, *p* = 0.036; ρG = −0.29, *p* = 0.043; ρE = 0.11, *p* > 0.05) and thalamic radiation (*r* = −0.13, *p* = 0.005; ρG = −0.31, *p* = 0.043; ρE = 0.06, *p* > 0.05). The strongest evidence of a genetic relationship between BMI and regional fractional anisotropy was in the superior fronto-occipital fasciculus (*r* = −0.13, *p* = 0.005; ρG = −0.39, *p* = 0.020; ρE = 0.12, *p* > 0.05). No other effects were significant for BMI or WC.

A large portion of intersubject variance in fractional anisotropy of the superior fronto-occipital fasciculus and WC were influenced by shared genetic factors (*r* = −0.10, *p* = 0.085; ρG = −0.39, *p* = 0.030; ρE = 0.18, *p* > 0.05). No other effects were significant.

## Discussion

Shared genetic variance between adiposity and integrity of cerebral white matter was studied in a large, well-characterized sample of Mexican American families in the Genetics of Brain Structure and Function Study. We indexed adiposity using the body mass index (BMI) and waist circumference (WC) measurements. Integrity of cerebral white matter was indexed using whole-brain average and regional fractional anisotropy measurements for ten major white matter tracts. We used the genetic correlation analysis to measure shared genetic variance between traits (Almasy et al., 1997) to assess the degree of shared genetic variance of adiposity with whole brain and regional measures of fractional anisotropy independently.

We found that BMI and WC shared 6 and 2%, respectively, of genetic variation in global and up to 15% variation in region-specific fractional anisotropy values. The highest shared genetic variance was observed between fractional anisotropy and BMI/WC in the superior fronto-occipital fasciculus (15%) and between BMI in the internal capsule (8%), thalamic radiation (10%), and sub-regions of the corpus callosum (genu: 6%; body: 9%; splenium: 7%). Results suggest that the same genetic factors that are associated with higher BMI were linked to progressively lower fractional anisotropy values in these regions.

Evaluation of heritability estimates for two adiposity measurements and the fractional anisotropy revealed that additive genetic factors explained 58% and 57% of BMI and WC, respectively and nearly 50% of inter-subject variance in global fractional anisotropy. Population heritability estimates range from 40 to 70% (Barsh et al., 2000; Challis and Yeo, 2002), and heritability values of many obesity-related phenotypes that explain inter-subject variability by individual genetic factors falling within the same range (between 40 and 70%) (Hasselbalch, 2010). Similarly high heritability values are observed for structural brain phenotypes (Kochunov et al., 2010; Winkler et al., 2010; Chiang et al., 2011). These include white matter integrity, regional and total brain volumes (Posthuma et al., 2000; Hulshoff Pol et al., 2006), and cortical thickness (Thompson et al., 2001; Schmitt et al., 2008). Evidence from twin and adoption studies support the important role of genetic factors (Maes et al., 1997), demonstrating that genetic factors jointly influence up to 46% of phenotypic variation in indices of white matter integrity (Kochunov et al., 2010). Such heritabilities support the pursuit of information on the interplay of genes and environment to better determine those who are more likely to develop obesity in a given environment (Maes et al., 1997).

By-tract genetic correlation findings demonstrated that tract-wise fractional anisotropy values in the internal capsule, thalamic radiation, genu, body, and splenium of the corpus callosum and the superior fronto-occipital fasciculus shared a significant proportion of genetic variance with BMI. The highest genetic correlation was observed between adiposity measurements and the superior fronto-occipital fasciculus. This is a long tract that carries axons interconnecting frontal, occipital, and the posterior temporal and parietal lobes. To our knowledge, no previous relationships have been reported between obesity and white matter deficits in this region. However, an examination of a younger cohort of females with anorexia nervosa by Kazlouski et al. (2011) demonstrated reduced FA in both the cingulum and fronto-occipital fasciculus among underweight participants compared to females in a healthy weight range. Findings of reduced bilateral fractional anisotropy in anorexia nervosa patients compared to controls have also been reported in the posterior thalamic radiation (Frieling et al., 2012), which connects the thalamus with the occipital and parietal lobes through cerebral white matter regions including the posterior limb of the internal capsule. Findings in patients with anorexia are in line with our results that a large portion of intersubject variance in BMI and fractional anisotropy of the posterior thalamic radiation and internal capsule were influenced by shared genetic factors. Occipitotemporal and frontal results may suggest relationships between distorted body image and white matter alterations connecting regions involved in body image perception (Frieling et al., 2012). It is possible that white matter changes are not directly due to weight gain in obesity, and are instead a symptom of behavior common to both obesity and eating disorders such as impaired impulse control, drastic changes in eating habits, or distorted body perception.

In addition to the fronto-occipital fasciculus, we observed significant decline in sub-divisions of the corpus-callosum. Corpus callosum fibers traverse the left and right hemispheres, facilitating contralateral communication. Region-specific differences in the heritability of white matter integrity have previously been reported in the genu, splenium, frontal, parietal, and occipital regions (Pfefferbaum et al., 2001; Chiang et al., 2009). Our finding parallels previous reports that corpus callosum microstructure is under significant genetic control (Pfefferbaum et al., 2000, 2001). Findings among late life adults indicated joint genetic and environmental influence on the genu and splenium (Pfefferbaum et al., 2001), however, we did not find evidence that microstructural coherence of the corpus callosum was jointly influenced by genetic and non-genetic (i.e., environmental) factors in the population studied. We found no evidence of environmental correlations between BMI or WC and fractional anisotropy in any regions. Corresponding decline in white matter volume in the genu, splenium, and whole corpus callosum are documented among obese adults (Stanek et al., 2011; Xu et al., 2013). One mechanism by which the integrity of the cerebral white matter is influenced may be via the same genetic and epigenetic mechanisms that lead to metabolic dysregulation and development of obesity.

It is of interest the rate of obesity in our sample (51%) was higher than 2009–2010 National Health and Nutrition Examination Survey (NHANES) rates for Mexican American men (37%) and women (45%) (Flegal et al., 2012). It is feasible that a large portion of the variation in adult bodyweight in the current energy-rich environment is due to genetic factors, albeit allowed to thrive due to the presence of environmental triggers. Epigenetic research posits that the human genome responds to environmental changes through altered gene expression, potentially influencing obesity. Chemicals (e.g., food) in the environment trigger epigenetic changes in hundreds of genes in the brain (Kumar, 2008; Kochunov et al., 2013) potentially influencing white matter fractional anisotropy with changes in body weight. This hints at a potential gene-environment interface, in which environmentally-triggered alterations to genes in the brain that may be associated with obesity become ingrained in the genome and passed on to future generations. If paired with overeating and/or sedentary behavior the risk of continued increases in adiposity rises, particularly among genetically-linked communities.

Genetic factors impact white matter development, which may subsequently impair decision-making and increase risk of diet-associated weight gain. Alternatively, metabolic dysregulation due to hormonal activity of the adipose tissue is considered to be a potential culprit (Despres, 2006). Visceral adipose tissue, in particular, is a key correlate of metabolic abnormalities present in obesity and produces inflammatory molecules which promote insulin resistance (Despres, 2006). WC is considered the best marker of abdominal visceral fat (Pouliot et al., 1994) and has been used as an index of diabetes prevalence among Mexican Americans in San Antonio (Lorenzo et al., 2007). Reductions in WC resulting from weight loss may be a promising means of improving white matter deficits, particularly in this population.

## Conclusions

Genetics and environment contribute to intersubject variance in obesity-related phenotypes and to white matter integrity (Stoeckel et al., 2008; Hasselbalch, 2010; Chiang et al., 2011) but the mechanisms of the genetic effects remain poorly understood. Our data demonstrate a shared genetic variance among phenotypic differences in obesity and brain integrity; however, the precise pathophysiology of obesity is unknown. Individual differences in food preferences, as a consequence of either genetic or experiential factors, may increase one's vulnerability to overeat when placed in a food-rich setting. Whereas environmental influences that can override factors involved in satiety are known to play a role (Stoeckel et al., 2008), there is a substantial genetic component to white and gray matter volume (Hulshoff Pol et al., 2006) and measures of obesity (Hasselbalch, 2010).

The present study sample was much larger than previous published studies and confirms that excess weight is associated with region-specific frontal and occipitotemporal white matter deficits in adults across a wide age range. This is an important first step to uncovering the mechanism for obesity-associated deficits in brain integrity. While advanced statistical genetic methods for family-based data allow for the formal detection of such interactions within cross-sectional data, longitudinal family studies will be required to establish if there is any causality between the two genetically influenced variables. Understanding the influence of shared genetic and environmental factors on phenotypic variation in white matter deficits and obesity could hold promise for future preventive and therapeutic strategies to combat obesity.

## Funding

This work was supported by a grant from the National Institute for Heart, Lungs and Blood (HL045522) to John Blangero. This research was also supported by the National Institute of Biomedical Imaging and Bioengineering (EB01561) to Peter Kochunov, the National Institute of Mental Health (MH078111) to John Blangero, (MH077230) to Laura M. Rowland, and (MH078143, MH083824) to David C. Glahn and the National Institute of Diabetes and Digestive and Kidney Diseases (DK082610) to Joanne E. Curran. SOLAR is supported by National Institute of Mental Health grant (MH059490) to John Blangero. Parts of this investigation were conducted in facilities constructed with support from the Research Facilities Improvement Program (Grant Number C06 RR013556, RR017515) from the National Center for Research Resources, National Institutes of Health. The AT&T Genomics Computing Center supercomputing facilities used for this work were supported in part by a gift from the AT&T Foundation and with support from the National Center for Research Resources (Grant Number S10 RR029392).

### Conflict of interest statement

The authors declare that the research was conducted in the absence of any commercial or financial relationships that could be construed as a potential conflict of interest.
